# Nelarabine-associated rhabdomyolysis with severe creatine kinase elevation in a pediatric patient with T-cell acute lymphoblastic leukemia: a case report

**DOI:** 10.3389/fonc.2026.1901440

**Published:** 2026-08-05

**Authors:** Fulden Aycan, Anna E. Spykerman, Christopher Carter Barnett

**Affiliations:** Stead Family Department of Pediatrics, Carver College of Medicine, The University of Iowa, Iowa City, IA, United States

**Keywords:** chemotherapy toxicity, nelarabine, pediatric, rhabdomyolysis, T-ALL, T-cell acute lymphoblastic leukemia

## Abstract

Nelarabine is a purine nucleoside analog used in relapsed or refractory T-cell acute lymphoblastic leukemia (T-ALL) and in up-front pediatric therapy. Although neurologic toxicity is dose-limiting, skeletal muscle toxicity, including rhabdomyolysis, has been reported. Rhabdomyolysis occurred in approximately 2% of nelarabine-treated patients in pediatric COG AALL0434, although the clinical features of these events were not detailed. We report a 7-year-old patient with T-ALL who developed rapidly progressive diffuse myalgia, muscle stiffness, impaired ambulation, dark urine, presumptive myoglobinuria, and a peak creatine kinase (CK) level exceeding 70,000 U/L shortly after nelarabine administration during consolidation. Nelarabine was discontinued, and aggressive intravenous hydration led to complete clinical and biochemical recovery without acute kidney injury or renal replacement therapy. This case provides a detailed pediatric description of clinically overt nelarabine-associated rhabdomyolysis and emphasizes early recognition, renal-protective management, and CK monitoring in symptomatic patients.

## Introduction

Nelarabine is a purine nucleoside analog approved for relapsed or refractory T-cell acute lymphoblastic leukemia (T-ALL) and lymphoblastic lymphoma (T-LLy) and is also incorporated into up-front therapy for pediatric T-ALL. It is a prodrug converted by adenosine deaminase to 9-β-D-arabinofuranosylguanine (ara-G), which is subsequently phosphorylated intracellularly to the active metabolite ara-G triphosphate (ara-GTP). Accumulation of ara-GTP inhibits DNA synthesis and promotes cell death (1–3). Its toxicity profile is primarily characterized by dose-limiting neurotoxicity; however, post-marketing reports have also described skeletal muscle toxicity, including rhabdomyolysis with marked CK elevation (2, 3).

The mechanism of nelarabine-associated skeletal muscle toxicity remains poorly understood, although direct myocellular toxicity has been proposed. Rhabdomyolysis was reported in approximately 2% of nelarabine-treated patients in the pediatric COG AALL0434 trial (4, 5). However, the published trial report provided aggregate adverse-event data and did not describe the associated symptoms, degree of CK elevation, presence of myoglobinuria, renal complications, or management. Therefore, the proportion of events representing isolated or minimally symptomatic CK elevation versus clinically overt rhabdomyolysis cannot be determined. Detailed descriptions of clinically overt pediatric rhabdomyolysis remain limited, while published case reports have predominantly involved adults (6–8). Recognition of this toxicity is clinically important, particularly in patients with additional renal vulnerability. We report a pediatric patient with T-ALL who developed severe, clinically overt rhabdomyolysis shortly after nelarabine administration.

## Case description and diagnostic assessment

A 7-year-old male with T-cell acute lymphoblastic leukemia, diagnosed in March 2026, was receiving consolidation chemotherapy according to the Children’s Oncology Group AALL0434 protocol. His prior oncologic course was notable for hyperleukocytosis at diagnosis and tumor lysis syndrome complicated by dense anuric stage 3 acute kidney injury requiring continuous renal replacement therapy and temporary hemodialysis, followed by renal recovery. During recovery, he experienced electrolyte wasting and intermittent hematuria and proteinuria. Additional comorbidities included hypertension treated with amlodipine and hydrochlorothiazide and metabolic acidosis treated with sodium citrate/citric acid. His concomitant medications included trimethoprim-sulfamethoxazole, fluconazole, gabapentin, calcium carbonate, cholecalciferol, ondansetron, and olanzapine. The patient completed standard-of-care induction chemotherapy per COG AALL1231 Arm A utilizing dexamethasone 3 mg/m^2^ twice daily for days 1–28, vincristine 1.5 mg/m^2^ weekly on days 1, 8, 15, and 22, daunorubicin 25 mg/m^2^ weekly on days 1, 8, 15, and 22, and calaspargase pegol 2,500 IU/m^2^ once on day 4. He was found to be MRD-negative at the end of induction. He completed induction chemotherapy approximately 13 days prior to starting consolidation therapy. He did not receive any known myotoxic medications within the 13 days between the end of the induction phase and the beginning of the consolidation phase.

## Chemotherapy exposure and symptom onset

The patient started consolidation chemotherapy per COG AALL0434, Arms B/D, with nelarabine approximately 13 days after completion of his induction phase. He received IV nelarabine 650 mg/m^2^ daily for days 1–5. After completion of his 5th day of nelarabine, he was clinically stable with only mild intermittent bilateral upper-extremity soreness, which was felt to be a residual effect of his corticosteroid induction course. He had no fever, neurologic deficits, or functional limitations. Within 12 h of his 5th nelarabine infusion, he developed progressively worsening diffuse myalgia, initially affecting both upper extremities and subsequently the lower extremities and hands. On consolidation day 6, symptoms progressed to severe generalized muscle stiffness, pain that limited mobility, and dark tea-colored urine. There were no reported trauma, strenuous exercise, hyperthermia, seizure, prolonged immobilization, recent clinical infection, or known sick contact.

## Admission and evaluation

Given concern for chemotherapy-associated muscle toxicity, he was admitted for evaluation. On admission, he was afebrile and hemodynamically stable. Physical examination demonstrated generalized discomfort with movement, bilateral calf tenderness, mild extremity fullness, abdominal distention without tenderness, and an antalgic (“duck”) gait caused by myalgia. Neurologic examination remained normal throughout hospitalization without focal weakness, altered mental status, or paresthesia. Urine collected on admission appeared dark brown.

Initial laboratory evaluation demonstrated a CK level of 61,791 U/L on admission, which peaked at 91,717 U/L on hospital day 3. Urinalysis demonstrated 2+ blood with 0–2 red blood cells per high-power field on microscopy, supporting presumptive myoglobinuria rather than true hematuria; however, urine or serum myoglobin was not measured directly. Additional urinary findings included transient 3+ proteinuria with an elevated urine protein-to-creatinine ratio of 4.54 on admission. Urine neutrophil gelatinase-associated lipocalin (NGAL) was initially elevated at 143 ng/mL. Serial potassium, phosphorus, and total calcium levels remained within normal limits throughout hospitalization. Ionized calcium, CK-MB, and troponin were not measured. The patient had no fever, respiratory symptoms, gastrointestinal symptoms, known sick contacts, or other clinical findings suggestive of an acute viral infection. A respiratory viral panel, viral serologic testing, and inflammatory markers such as C-reactive protein were not obtained; therefore, viral myositis could not be definitively excluded.

## Diagnostic assessment and therapeutic intervention

The differential diagnosis included nelarabine-associated myotoxicity, viral or inflammatory myositis, electrolyte-mediated muscle injury, trauma or exertion, seizure-related muscle injury, an underlying metabolic myopathy, and myotoxicity from concomitant medications. Trauma, exertion, seizure-related injury, and electrolyte-mediated injury were considered less likely based on the clinical history and normal serial potassium, phosphorus, and total calcium levels. Trimethoprim-sulfamethoxazole, fluconazole, and olanzapine were considered potential medication-related contributors, although each had been administered before the event without prior muscle symptoms. The close temporal relationship between nelarabine administration and symptom onset, together with improvement after nelarabine withdrawal, supported probable nelarabine-associated nontraumatic rhabdomyolysis, while alternative causes could not be excluded with certainty.

Causality was assessed using the Naranjo adverse drug reaction probability scale. The patient received 1 point for previous reports of nelarabine-associated rhabdomyolysis, 2 points because the reaction occurred after nelarabine administration, 1 point because the clinical and biochemical abnormalities improved after nelarabine was discontinued, and 1 point because the reaction was supported by objective findings, including marked CK elevation and presumptive myoglobinuria. No points were assigned for rechallenge, placebo exposure, toxic drug concentrations, a dose-response relationship, or a similar previous reaction. Because alternative causes could not be excluded with certainty, the alternative-cause item was assigned 0 points.

Nelarabine was discontinued, and intravenous isotonic fluids were initiated at 1.5 times maintenance and increased to twice maintenance because CK continued to rise. Strict intake and output measurement, daily weights, serial CK testing, and twice-daily metabolic panels were performed. Nephrology recommended high-rate hydration, close renal and electrolyte surveillance, avoidance of nephrotoxic agents, continuation of sodium citrate, and consideration of urine alkalinization or diuresis if clinically indicated. A single dose of furosemide was later administered for mild positive fluid balance. Renal replacement therapy was not required. Serial neurologic examinations remained normal throughout hospitalization, with no evidence of nelarabine-associated encephalopathy or peripheral neuropathy.

Pain was treated with acetaminophen, brief opioid therapy, and continuation of home gabapentin; nonsteroidal anti-inflammatory drugs were avoided because of renal risk. Chemotherapy was temporarily held, and the next treatment cycle was delayed by one week pending clinical and biochemical recovery.

## Clinical course and outcome

**Figure 1:**
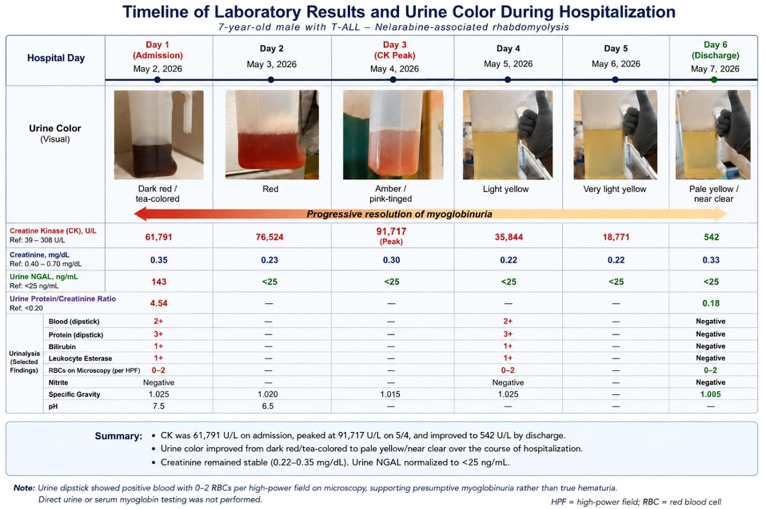
Clinical and laboratory course of nelarabine-associated rhabdomyolysis.

CK increased during the first 48 h, peaked at 91,717 U/L on hospital day 3, and subsequently declined in parallel with improvement in myalgia, muscle stiffness, mobility, and urine color. By hospital days 4–5, the patient had near-complete resolution of pain and was ambulating without difficulty. Intravenous fluids were gradually reduced from twice maintenance to 1.5 times maintenance as CK declined. Serial potassium, phosphorus, and total calcium levels remained within normal limits, without hyperkalemia, hyperphosphatemia, hypocalcemia, or rebound hypercalcemia during the observed recovery period. CK-MB and troponin were not measured; however, the patient had no chest pain, hemodynamic instability, or other clinical findings suggestive of cardiac involvement. Serial urine color changes and laboratory trends throughout hospitalization are shown in **Figure 1**.

Urine NGAL decreased from 143 ng/mL on admission to <25 ng/mL on hospital day 4 and remained <25 ng/mL on hospital day 5. At discharge on hospital day 6, CK had decreased to 542 U/L, urine color had improved from dark red or tea-colored to pale yellow or nearly clear, and the urine protein-to-creatinine ratio had decreased from 4.54 to 0.18. Despite the severity of rhabdomyolysis and the patient’s prior history of CRRT-requiring acute kidney injury, serum creatinine remained stable at 0.22 mg/dL–0.35 mg/dL, with preserved urine output and no evidence of recurrent acute kidney injury.

The patient was discharged home in stable condition with complete neurologic recovery, preserved renal function, and no residual weakness. Follow-up with pediatric hematology/oncology and nephrology was arranged within 7 days of hospital discharge for continuation of chemotherapy and monitoring of renal function, respectively.

## Discussion

Rhabdomyolysis is a clinical syndrome caused by skeletal muscle injury and the release of intracellular muscle components into circulation. Common manifestations include myalgia, muscle tenderness, swelling, weakness, and dark urine. Although rhabdomyolysis frequently occurs after trauma or crush injury, other causes include infections, medications, toxins, hyperthermia, seizures, prolonged immobilization, electrolyte disturbances, and genetic or inflammatory muscle disorders. Creatine kinase is the most sensitive laboratory marker and typically begins to rise within 12 h of muscle injury, peaks within 24 h–72 h, and declines over several days after the injury has ceased (9–11). Severe rhabdomyolysis may result in hyperkalemia, hyperphosphatemia, early hypocalcemia, rebound hypercalcemia during recovery, and pigment-associated acute kidney injury.

Nelarabine-associated skeletal muscle toxicity appears to occur along a spectrum ranging from isolated or minimally symptomatic CK elevation to clinically overt rhabdomyolysis with myalgia, myoglobinuria, electrolyte disturbances, acute kidney injury, and the need for renal replacement therapy. Rhabdomyolysis was reported in approximately 2% of patients receiving nelarabine during consolidation therapy in the pediatric COG AALL0434 trial (4, 5). However, the published reports provided limited patient-level information regarding associated symptoms, degree of CK elevation, presence of myoglobinuria, renal involvement, treatment, and outcomes. Therefore, the proportion of reported events representing isolated biochemical abnormalities versus a complete clinical syndrome cannot be determined. The present case provides a detailed description of clinically overt pediatric rhabdomyolysis characterized by rapidly progressive diffuse myalgia and stiffness, impaired mobility, dark urine with urinalysis findings supporting presumptive myoglobinuria, and a peak CK of 91,717 U/L.

Detailed case reports of nelarabine-associated rhabdomyolysis remain limited and have predominantly involved adults (6–8). Haider et al. and Utsumi et al. described grade 4 CK elevations without substantial electrolyte abnormalities or renal injury (6, 8), whereas Pandey et al. reported severe rhabdomyolysis complicated by hyperkalemia, hyperphosphatemia, acute kidney injury, and the need for emergent dialysis (7). Our patient had a history of tumor lysis syndrome-associated stage 3 acute kidney injury requiring continuous renal replacement therapy, creating particular concern for recurrent renal injury. Nevertheless, renal function remained preserved despite a peak CK of 91,717 U/L. The case is further distinguished by rapid symptom onset, impaired ambulation, presumptive myoglobinuria, and complete clinical and biochemical recovery following early nelarabine withdrawal and aggressive intravenous hydration.

The mechanism of nelarabine-associated skeletal muscle toxicity remains uncertain. Nelarabine is a prodrug that is rapidly converted by adenosine deaminase to 9-β-D-arabinofuranosylguanine, which is subsequently phosphorylated intracellularly to its active triphosphate form. Accumulation of the active metabolite inhibits DNA synthesis and produces cytotoxicity (1, 3, 12). Direct myocellular toxicity has been proposed, but preferential accumulation of nelarabine or its metabolites in skeletal muscle has not been established. Although nucleoside transporters may influence intracellular uptake of purine analogs, a specific role for transporters such as hENT1 or hCNT2 in nelarabine-associated muscle injury remains speculative.

Nelarabine and its metabolites undergo renal elimination, and reduced ara-G clearance has been reported in patients with renal impairment (12). It is therefore biologically plausible that impaired renal function could increase systemic drug exposure. However, nelarabine or ara-G concentrations were not measured in this patient, and renal function had recovered before nelarabine administration. His previous severe acute kidney injury may have increased his vulnerability to renal complications from rhabdomyolysis, but whether it contributed to the development of myotoxicity cannot be determined.

Causality was assessed using the Naranjo adverse drug reaction probability scale (13). The patient received one point for previous reports of nelarabine-associated rhabdomyolysis, two points because the reaction occurred after nelarabine administration, one point because the clinical and biochemical abnormalities improved after nelarabine withdrawal, and one point because the reaction was supported by objective findings, including marked CK elevation and presumptive myoglobinuria. No points were assigned for rechallenge, placebo exposure, toxic drug concentrations, a dose–response relationship, or a similar previous reaction. Because alternative causes could not be excluded with certainty, the alternative-cause item was assigned 0 points. The total Naranjo score was 5, indicating a probable rather than a definite adverse drug reaction.

Potential alternative contributors included trimethoprim-sulfamethoxazole, fluconazole, and olanzapine, each of which has rarely been associated with muscle injury. However, these medications had been administered for several weeks without previous muscle symptoms. Fluconazole-associated rhabdomyolysis has also been described primarily in the setting of interactions with statins, which the patient was not receiving. Viral myositis could not be definitively excluded because a respiratory viral panel, viral serologic testing, and inflammatory markers such as C-reactive protein were not obtained. Nevertheless, the patient remained afebrile and had no known sick contacts, respiratory symptoms, gastrointestinal symptoms, or other clinical findings suggesting an acute viral infection. Trauma, strenuous exercise, seizure, prolonged immobilization, hyperthermia, and electrolyte-mediated muscle injury were considered less likely based on the clinical history and normal serial levels of potassium, phosphorus, and total calcium. Thus, the temporal relationship to nelarabine and improvement after withdrawal support nelarabine as the probable cause, although definitive causality cannot be established.

Urine NGAL was monitored because of the patient’s history of severe acute kidney injury and the risk of pigment-associated tubular injury. NGAL is an early biomarker of renal tubular stress that may increase before serum creatinine in pediatric acute kidney injury, although it is not specific to rhabdomyolysis and is not routinely used in all centers (14). In this patient, urine NGAL decreased from 143 ng/mL on admission to <25 ng/mL on hospital day 4 and remained <25 ng/mL on hospital day 5, while serum creatinine and urine output remained stable. These findings were reassuring against progressive tubular injury but should be interpreted as supportive rather than diagnostic evidence.

This case report has several limitations. Causality was classified as probable rather than definitive, and nelarabine was not re-challenged. The primary oncology team for this patient chose to omit further doses of nelarabine. The decision to omit further nelarabine was the product of conversations at a multi-institutional pediatric leukemia and lymphoma tumor board, where expert opinion was to withhold further doses of nelarabine given the anecdotal concern that further administration may lead to subsequent and potentially worse episodes of rhabdomyolysis. Viral testing and inflammatory markers, including C-reactive protein, were not obtained because the patient lacked fever, known sick contacts, respiratory symptoms, gastrointestinal symptoms, or other clinical findings suggestive of acute infection. Consequently, viral myositis could not be definitively excluded and remains a limitation of this report. The patient was not evaluated for an underlying metabolic myopathy, such as a glycogen storage disorder or carnitine palmitoyltransferase II deficiency. Urine or serum myoglobin was not directly measured, and myoglobinuria was inferred from dark urine and positive dipstick blood with minimal red blood cells on microscopy. Ionized calcium, CK-MB, troponin, and nelarabine or ara-G concentrations were not measured. Although serial potassium, phosphorus, and total calcium levels remained normal, the absence of ionized calcium testing limits complete assessment of calcium disturbances. Long-term follow-up regarding recurrence, late renal effects, and subsequent nelarabine exposure was also limited at the time of this report.

After discharge from his hospitalization, our patient continued with and completed consolidation chemotherapy per COG AALL0434 with plans to omit further nelarabine doses. He tolerated all doses of IV cyclophosphamide (1,000 mg/m^2^/dose on days 8 and 50), IV/SQ cytarabine (75 mg/m^2^/dose on days 8–11, 15–18, 50–53, and 57–60), oral mercaptopurine (60 mg/m^2^/dose on days 8–21 and 50–63), and IV vincristine (1.5 mg/m^2^/dose on days 22, 29, 64, and 71). He experienced a grade III hypersensitivity reaction to IV calaspargase pegol (2,500 IU/m^2^/dose on day 22) requiring transition to IM asparaginase Erwinia chrysanthemi-rywn per institutional protocol. He has since tolerated all doses of IM asparaginase Erwinia to replace calaspargase pegol doses scheduled for days 22 and 64. He is due to start interim maintenance per COG AALL0434 and remains clinically well.

Rhabdomyolysis is a rare, underrecognized, and potentially life-threatening adverse effect of nelarabine that can present on a spectrum ranging from mild CK elevation to the need for emergent renal replacement therapy. Pediatric clinicians should maintain a high index of suspicion in patients presenting with myalgias and discolored urine shortly after exposure. Early identification is critical, as prompt intravenous hydration can prevent progression to renal failure. In patients receiving nelarabine as part of their T-ALL therapy, careful monitoring of musculoskeletal symptoms and CK levels should be considered practiceto prevent the life-threatening adverse effects of nelarabine-associated rhabdomyolysis.

## Data Availability

The original contributions presented in the study are included in the article/supplementary material. Further inquiries can be directed to the corresponding authors.

## References

[1] DeAngelo DJ . Nelarabine for the treatment of patients with relapsed or refractory T-cell acute lymphoblastic leukemia or lymphoblastic lymphoma. Hematol Oncol Clin North Am. (2009) 23:1121–35:vii–viii. doi: 10.1016/j.hoc.2009.07.008 19825456

[2] US Food and Drug Administration . Nelarabine Prescribing Information (2025). Available online at: https://www.accessdata.fda.gov/drugsatfda_docs/label/2025/021877Orig1s014lbl.pdf (Accessed May 19, 2026).

[3] Shimony S DeAngelo DJ Luskin MR . Nelarabine: when and how to use in the treatment of T-cell acute lymphoblastic leukemia. Blood Adv. (2024) 8:23–36. doi: 10.1182/bloodadvances.2023010303 37389830 PMC10784681

[4] Dunsmore KP Winter SS Devidas M Wood BL Esiashvili N Chen Z . Children’s Oncology Group AALL0434: a phase III randomized clinical trial testing nelarabine in newly diagnosed T-cell acute lymphoblastic leukemia. J Clin Oncol. (2020) 38:3282–93. doi: 10.1200/JCO.20.00256 32813610 PMC7526719

[5] Winter SS Dunsmore KP Devidas M Wood BL Esiashvili N Chen Z . Improved survival for children and young adults with T-lineage acute lymphoblastic leukemia: results from the Children’s Oncology Group AALL0434 methotrexate randomization. J Clin Oncol. (2018) 36:2926–34. doi: 10.1200/JCO.2018.77.7250 30138085 PMC6366301

[6] Utsumi A Goto Y Suzuki T Imai C Matsui S Sakaida E . Nelarabine-induced rhabdomyolysis in a patient with T-cell acute lymphoblastic leukemia: a case report. J Pharm Health Care Sci. (2022) 8:17. doi: 10.1186/s40780-022-00247-w 35690835 PMC9188041

[7] Pandey RK DiPippo A Kadia T Pemmaraju N Workeneh BT Jabbour E . Nelarabine-related rhabdomyolysis in a patient with T-cell acute lymphoblastic leukemia. Leuk Lymphoma. (2020) 61:2775–7. doi: 10.1080/10428194.2020.1791852 32654562

[8] Haider M Rizvi SA Kasi PM . Nelarabine-associated myotoxicity and rhabdomyolysis. Case Rep Hematol. (2015) 2015:825670. doi: 10.1155/2015/825670 25866685 PMC4381549

[9] Barbano B Sardo L Gasperini ML Gigante A Liberatori M Di Lazzaro GG . Drugs and rhabdomyolysis: from liver to kidney. Curr Vasc Pharmacol. (2015) 13:725–37. doi: 10.2174/1570161113666150130151839 25633322

[10] Zimmerman JL Shen MC . Rhabdomyolysis. Chest. (2013) 144:1058–65. doi: 10.1378/chest.12-2016 24008958

[11] Rout P Chippa V Adigun R . Rhabdomyolysis. In: Statpearls. StatPearls Publishing, Treasure Island, FL (2026). 28846335

[12] Buie LW Epstein SS Lindley CM . Nelarabine: a novel purine antimetabolite antineoplastic agent. Clin Ther. (2007) 29:1887–99. doi: 10.1016/j.clinthera.2007.09.002 18035189

[13] Naranjo CA Busto U Sellers EM Sandor P Ruiz I Roberts EA . A method for estimating the probability of adverse drug reactions. Clin Pharmacol Ther. (1981) 30:239–45. doi: 10.1038/clpt.1981.154 7249508

[14] Zou Z Chen B Tang F Li X Xiao D . Predictive value of neutrophil gelatinase-associated lipocalin in children with acute kidney injury: a systematic review and meta-analysis. Front Pediatr. (2023) 11:1147033. doi: 10.3389/fped.2023.1147033 37051429 PMC10083323

